# Comparison of levofloxacin versus clarithromycin efficacy in the eradication of Helicobacter pylori infection

**Published:** 2016

**Authors:** Ali Akbar Haji-Aghamohammadi, Ali Bastani, Arash Miroliaee, Sonia Oveisi, Saeed Safarnezhad

**Affiliations:** 1 Department of Internal Medicine,Velayat Clinical Research Development Unit, Qazvin University of Medical Sciences, Qazvin, Iran.; 2 Metabolic Diseases Research Center, Qazvin University of Medical Sciences, Qazvin, Iran.

**Keywords:** Helicobacter pylori, Dyspepsia, Peptic ulcer, Rapid urease test, Stool antigen

## Abstract

**Background:**

Helicobacter pylori (H.pylori) infection causes multiple upper gastrointestinal diseases but optimal therapeutic regimen which can eradicate infection in all the cases has not yet been defined. This study was designed to evaluate the efficacy of triple levofloxacin-based versus clarithromycin-based therapy.

**Methods:**

In this open-label randomized clinical trial study 120 patients who had esophagogastroduodenoscopy (EGD) with positive rapid urease test (RUT) were enrolled and divided into 2 groups. Case group was treated with levofloxacin (500 mg daily) plus amoxicillin (1 gr twice a day) plus omeprazole (20 mg daily) for 2 weeks. Control group was treated with clarithromycin (500 mg twice a day) plus omeprazole (20 mg daily) for 2 weeks. After the main course of treatment, they received maintenance treatment with omeprazole for 4 weeks. Stool antigen test was performed on them after two weeks of not having any medicine.

**Results:**

H.pylori eradication (intention to treat analysis) was successful in 75% of case group and 51.7% of control group showing a significant difference (P=0.008). H.p infection eradication (per-protocol analysis) was successful in 80.4% in case group and 57.4%% in control group showing significant difference (P=0.009). Drugs adverse effects causing discontinuation treatment were seen in 5% of case group and 3.3% of control group which have not shown a significant difference between the two groups (P=0.648).

**Conclusion:**

Triple therapy with levofloxacin-based regimen has better efficacy than clarithromycin-based regimen and as safe as it is.

The presence of organisms was first observed more than 100 years ago and their association with gastritis has been recognized since the 1970s (1). The true implication of these microbes was not fully realized, however, until 1982 when Marshall and Warren identified. Campylobacter pyloridis on culture, which was reclassified later it as H.pylori (2). H.pylori is the most common chronic bacterial infection in humans (3, 4). It has been demonstrated worldwide and in individuals of all ages. Conservative estimates suggest that 50 percent of the world population is affected. Infection is more frequent and acquired at an earlier age in the developing countries compared with industrialized nations (4). This organism is now known to cause chronic gastritis, dyspepsia, most peptic ulcers, gastric cancers and lymphoma. Hence, eradication of H.pylori can control or cure such diseases (5). Multiple regimens have been evaluated for H.pylori infection therapy in randomized controlled trials (6-10). Despite the numerous studies, the optimal therapeutic regimen has not yet been defined.

An appropriate effective regimen should be considered with regard to cost, side effects, and ease of administration. Triple therapy with a proton pump inhibitor (PPI), amoxicillin and clarithromycin is the most commonly recommended for first line treatment of H.p and defined as standard protocol (11-13). However, resistant types of H.p to this regimen are rising (5, 14, 15) and we need to evaluate more potent and available drugs in the first line treatment of H.p. Previously, levofloxacin as a family member of flourquinolones was effective and safe for second line and rescue therapy in eradication of H.p (16-18) but there are little experiments about the efficacy of this drug as the first line treatment for H.p infection. Also, with the consideration of high prevalence of H.p infection in Iran that were reported over 90% (19, 20) we decided to design the current study to evaluate the efficacy of triple levofloxacin-based versus clarithromycin based therapy.

## Methods

This study was a single center open label randomized clinical trial to compares the efficacy of levofloxacin versus clarithromycin in the eradication of H.p infection. Our primary end point was the drugs side effects and secondary end point was H.p infection eradication. This study was done under the supervision of the Ethics Committee of Qazvin University of Medical Sciences (reference number: 9943) and also was registered in Iranian Registry of clinical Trials (IRCT registration number: IRCT2015081818124N2). 

The participants of this study were selected consecutively from patients who had upper gastrointestinal symptoms and had undergone esophagogastroduodenoscopy (EGD) in Velayat Hospital in Qazvin city, central Iran over a 9-month period February 20 2015 to October 22 2015.

Inclusion criteria for this study were as follows: all patients undergoing upper endoscopic procedure (EGD) with positive rapid urease test (RUT) aged between 18 and 70 years old. RUT was applied to biopsies taken from the stomach antrum mucosa, to identify the presence of infection. RUT was considered positive if reagent tube color containing biopsy sample changed to red during the 24 hours after EGD. Exclusion criteria for this study were as follows: pregnancy, previous consumption of proton pump inhibitors (PPI) or bismuth and antibiotics in the preceding month, gastrointestinal (GI) bleeding or any complication of peptic ulcer disease (PUD), consumption of alcohol or substance; advanced chronic disease such as diabetes mellitus, hypertension, cirrhosis, cerebrovascular attack, renal insufficiency, coronary artery disease and gastric cancer, noncompliance to treatment or to complete duration treatment, coexistence of other conditions needing to consume another antibiotic or non-steroidal anti-inflammatory drugs (NSAIDs).

Two-hundred and thirteen patients were recruited and 120 patients entered to the study according to the inclusion criteria. After completing the consent form 120 patients were divided into 2 groups by balance block method (each group contained 60 patients). The case group was treated by levofloxacin (500 mg daily) plus amoxicillin (1 gr twice a day) plus Omeprazole (20 mg daily) for 2 weeks. The control group received clarithromycin (500 mg twice a day) plus Amoxicillin (1 g twice a day) plus omeprazole (20 mg daily) for 2 weeks. Maintenance treatment with omeprazole was continued for 4 weeks. Two weeks after the end of treatment period, all patients were tested by H.p infection by stool antigen test using monoclonal antibody against H.p antigen in collected stool. The stool antigen test was performed using the HpSA enzyme linked immunosorbent assay (ELISA) method (Premier Platinum HpSA, Meridian Diagnostics, Cincinnati, OH, USA).

 Baseline information, demographic features, clinical and endoscopy findings were recorded. Data regarding drugs adverse effects were provided 1 to 2 weeks after the start of treatment. At endpoint the two groups were compared with respect to proportion of H.p eradication. Stool examination was selected because of its cost effectiveness and availability. In addition, its diagnostic sensitivity and specificity are comparable with urease breath test at 94% and 92%, respectively (5). Data were analyzed with SPSS16 (SPSS Inc., Chicago, IL, USA). Chi-square test, or Fisher’s exact test were used in proportions and independent t-test was for utilized comparison of quantitative data. A P-value of 0.05 or less was considered statistically significant. All P-values were two-sided. The intention-to-treat (ITT) analysis included all patients who had taken one tablet of drugs. In the per-protocol analysis, the patients with severe drug side effects and those who lost follow-up examination were excluded.

## Results

**Figure 1:**
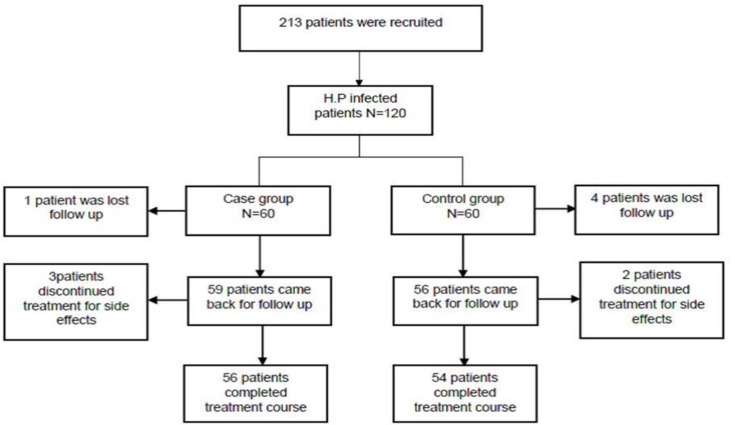
Schematic representation of groups in study

One-hundred and twenty patients were enrolled in two groups and each group contained 60 patients. In the case group, one patient was lost to follow-up and was excluded and in the control group, four patients were lost to follow-up and thus, were excluded. Also, during-follow-up, 3 patients in case group and 2 patients in control group discontinued treatment because of severe drug side effects (figure 1). The mean age of the patients was 43.3±13.85 years old in case group and 42.85±12.78 years old in the control group. There was no statistical difference between groups (P=0.775). Twenty seven (45%) patients in the case group and 26 (43.3%) were males in the control group. No significant difference was found between groups (P=0.854). Frequency of clinical features and endoscopy findings in both groups are shown in table 1. Severe side effects resulting to the discontinuation of treatment were seen in 3(5%) cases and in 2(3.6%) controls (P=1). Frequencies of mild and severe side effects were shown in table 2. H.p eradication rates for intention-to-treat analysis were observed in 45 (75%) cases versus 31(51.7%) controls (P=0.008). H.p eradication rates for per-protocol analysis were 45.56 (80.4%) in the case group and 31.54 (57.4%) in the control group. (P=0.009).

**Table 1:** Frequency of patient symptoms and EGD^†^ finding in both groups

**Groups** **Symptom**	**Clarithromycin** **(N=60)**	**Levofloxacin** **(N=60)**	**P value** *
Epigastrium pain	38(63.3%)	37(61.7%)	
Nonspecific abdominal pain	7(11.7%)	12(20%)	
Nausea	1(1.7%)	3(5%)	0.440
Bloating	11(18.3%)	8(13.3%)	
Early satiety	3(5%)	0(0%)	
**EGD** ^† ^ **Finding**			
Esophagitis	3 (5%)	4(6.7%)	
Antral erythema	29(48.3%)	22(36.7%)	
Gastro-duodenum erosion	7(11.7%)	10(16.7%)	
Gastric ulcer	6(10%)	9(15%)	0.740
Duodenal ulcer	4(5%)	2(3.3%)	
Esophagitis& Antral erythema	7(11.7%)	5(8.3%)	
Non ulcer dyspepsia (Normal EGD)	5(8.3%)	8(13.3%)	

* P value less than 0.05 is significant

† Esophagogastroduodenoscopy

**Table 2:** Side effects of treatment in both groups

**Groups** **Side effects**	**Clarithromycin** **(N=56)**	**Levofloxacin** **(N=59)**	**Pvalue**
Nausea	4(7.1%)	2(3.4%)	
Skin rash& itching	0(0%)	2(3.4%)	0.280
Diarrhea	3(5.4%)	1(1.7%)	
None	49(87.5%)	54(91.5%)	

* P value less than 0.05 is significant

## Discussion

Extensive efforts have been made to determine individuals with H.p infection requiring eradication. At present there is a broad consensus to eradicate H.p infection in peptic ulcer disease and dyspepsia (5). These patients were the main participants in our study.

In our study, eradication rate was 75% in case group and 51.7% in the control group according to intention- to-treat analysis. In addition eradication rate were 80.4% in case group and 57.4% in control group according to per-protocol analysis. These differences indicate that levofloxacin-based triple therapy is more effective than clarithromycin based therapy in the eradication of H.p infection. In an earlier study by Gopal et al. (21) on 74 patients with peptic ulcer perforation, H.p eradication rates were 69% and 79% in clarithromycin-based regimen versus 80% and 87% in levofloxacin-based regimen on intention to treat and per protocol analysis respectively.

In another study (14) of patients with dyspepsia, H.p infection eradication rate was 66.67% for clarithromycin triple therapy and 94.87% for levofloxacin, based triple therapy as first line regimens.

Similar results have been reported in a study of Chinese population (22) with eradication rates of 74.5% and 78.2% in clarithromycin triple therapy, versus 82.4% and 83% in levofloxacin based triple therapy regimen according to intention to treat and per protocol analysis respectively.

Discrepancies across various studies may be explained by overuse of antibiotic particularly flourquinolones in Iran as reported in previous studies (23-25). 

Perna et al. (26) suggest that levofloxacin resistance H.p maybe associated with prior flourquinolones consumption during the past ten years. In two systematic reviews in levofloxacin based rescue therapy for H.p eradication rate was 51.6%-94.3% (17, 18). However, variations in ethnicities, drug dosage and duration of therapy should be also considered for explaining different results. Studies from East Asia had lower eradication rate but studies in India and Western countries had higher eradication rates (14, 21, 17, 18). 

Over all in our study drugs side effects were seen in 8.5% of the patients in case group and 12.5% in the control group. Additionally, severe side effects causing discontinuation of treatment regiments were seen in 5% of patients in case group and in 3.6% of patients in control group which indicate levofloxacin as safe as clarithromycin. These findings are consistent with other studies (15, 17, 18). The cost of treatment may partly affect in the decision to select treatment regimen. Regarding higher cost of levofloxacin, this issue may prevent the use of levofloxacin as the first line of empiric treatment for H.p infection. This study has limitations regarding its insufficient sample size particularly to show significant adverse effects between the two groups. In addition, we did not collect data regarding the efficacy of treatment on clinical features of patients. 

In conclusion the results of this study indicate that levofloxacin-based triple therapy is preferable to claritromycin-based regimen in H.p eradication to achieve an optimal eradication rate of 80% or more as recommended in treatment guideline (5). However, the cost of treatment and the possible higher rate of severe reaction should be considered in the selection of this regimen.

## Notes

**Funding: **This study was funded by Qazvin University of Medical Sciences**.**
